# Prognostic Factors of Disease Recurrence in Breast Cancer Using Quantitative and Qualitative Magnetic Resonance Imaging (MRI) Parameters

**DOI:** 10.1038/s41598-020-64564-6

**Published:** 2020-05-05

**Authors:** Jeongmin Lee, Sung Hun Kim, Bong Joo Kang

**Affiliations:** Department of Radiology, Seoul St. Mary’s Hospital, College of Medicine, The Catholic University of Korea, Seoul, Republic of Korea

**Keywords:** Breast cancer, Cancer imaging

## Abstract

The purpose of this study was to investigate prognostic factors predicting recurrence of breast cancer, focusing on imaging factors including morphologic features, quantitative MR parameters, and clinicopathologic factors. This retrospective study was approved by our institutional review board, and the requirement to obtain informed consent was waived. A total of 267 patients with breast cancer were enrolled in this study, who underwent dynamic contrast-enhanced magnetic resonance imaging (MRI) before surgery from February 2014 to June 2016. Imaging parameters of MRI, including morphologic features, perfusion parameters, and texture analysis, were retrospectively reviewed by two expert breast radiologists. Clinicopathologic information of enrolled patients was also reviewed using medical records. Univariable and multivariable Cox proportional hazards regression analyses were used to identify factors associated with cancer recurrence. C statistics was used to discriminate low and high risk patients for disease recurrence. Using Kaplan-Meier survival analysis, disease-free survival was compared between patients who experienced recurrence and those who did not. At a median follow up of 49 months, 32 patients (12%) showed disease: six cases of ipsilateral breast or axilla recurrence, one case of contralateral breast recurrence, 24 cases of distant metastasis, and one case of both ipsilateral breast recurrence and distant metastasis. Of multiple imaging features and parameters, increased ipsilateral vascularity and higher positive skewness of texture analysis showed significant association with disease recurrence in every multivariable model regardless of tumor subtype and pathologic stage. Pathologic stage, especially if higher than stage II, showed significant association with disease recurrence and its highest hazard ratio was 3.45 [95% confidence interval (CI): 1.37–8.67, *p* = 0.008]. Of the multivariable models, the model including clinico-pathologic factors and both qualitative and quantitative imaging parameters showed good discrimination with a high C index value of 0.825 (95% CI: 0.755–0.896). In addition, recurrence associated factors were associated with short interval time to disease recurrence by Kaplan-Meier survival analysis. Therefore, comprehensive analysis using both clinico-pathologic factors and qualitative and quantitative imaging parameters is more effective in predicting breast cancer recurrence. Among those factors, higher pathologic stage, increased ipsilateral vascularity and higher positive skewness of texture analysis could be good predictors of breast cancer recurrence. Moreover, when these three factors are applied comprehensively, they may also be the predictors for poor survival.

## Introduction

Breast cancer is a leading cause of death among women worldwide and the second most common cancer in Korean women. However, with increased screening and development of treatment methods, breast cancer mortality rates have improved over the last few decades^1,2^. Postoperative adjuvant systemic therapies including chemotherapy, hormone therapy, and target agents have contributed to improving mortality rates in breast cancer patients^3–5^.

Despite such improvements, women who have previously undergone breast cancer treatment are at higher risk of breast cancer than those without history of breast cancer. In addition, patients with recurrent breast cancer have worse prognoses than those who have not recurred^6–8^. For this reason, many efforts to predict prognosis in breast cancer patients including those with cancer recurrence have been made.

Oncotype DX (Genomic Health, Redwood City, CA, USA), a commercially available 21-gene breast cancer recurrence score assay, was recently introduced to predict prognosis in early breast cancer (estrogen receptor-positive/human epidermal growth factor receptor 2-negative/lymph node-negative) patients^9^. Recurrence score, calculated according to Oncotype DX, can help determine the direction of treatment in early breast cancer patients who need adjuvant systemic therapy after surgery^10^. However, Oncotype DX is limited in indications and expensive, making it difficult to apply to all breast cancer patients. In fact, Oncotype DX is performed only in one-third of eligible breast cancer patients in United States, and less than 20% of patients in European countries^11,12^.

Breast MRI is performed in most patients with newly diagnosed breast cancer and can both diagnose breast cancer accurately and predict cancer prognosis using variable imaging features. Previous studies identified that several imaging features including rim enhancement pattern of tumor^13,14^, presence of peritumoral edema on T2-weighted image^15,16^, higher degree of background parenchymal enhancement (BPE), and increased vascularity around the tumor, indicate poor prognosis in breast cancer^13–17^. In addition to morphologic features of tumor, quantitative MRI parameters derived from advanced MR techniques have been recently developed for prediction of breast cancer prognosis. For example, perfusion parameters derived from dynamic contrast-enhanced MRI (DCE-MRI) and tumor heterogeneity based on texture analysis can predict cancer prognosis^18,19^. Such quantitative parameters may be more objective indicators than morphologic characteristics for predicting cancer prognosis.

Therefore, the purpose of this study is to investigate prognostic factors that predict breast cancer recurrence comprehensively, using MR morphologic features and quantitative MR parameters in addition to clinicopathologic factors.

## Results

### Patients

At a median follow up of 49 months (range 1 to 64 months), there were 32 cases (12%) of cancer recurrence (median 28.5 months, range 1 to 63 months): six cases of recurrence in the ipsilateral breast or axillary lymph node, one case in the contralateral side breast, 24 cases of distant metastases, and one case of recurrence both in the ipsilateral breast and distant metastasis. The median age of patients diagnosed with recurrent cancer was 48.5 (range, 25 to 74 years), and that of patients without disease recurrence was 51 years (range, 24 to 86 years), which were not significantly different (*p* = 0.468). Regarding immunohistochemical staining subtype, luminal B type was the most common subtype across all patients (124 of 267, 46.8%) as well as in the recurrence group (18 of 32, 56.3%). Regarding pathologic stage, stage II was the most common in the non-recurrent group (116 of 235, 49.6%), while stage III was most common in the recurrent group (12 of 32, 38.7%). Other characteristics of the 267 patients are summarized in Table 1.

**Table 1:** Patient characteristics.

	Total	Non-recurrent	Recurrence	p value
	(n = 267)	(n = 235)	(n = 32)	
**Patient age**				
mean ± SD.	50.7 ± 10.8	50.9 ± 10.7	49.6 ± 11.6	0.468
Median(IQR)	51.0 (43.0–58.0)	51.0 (43.0–58.0)	48.5 (41.5–56.5)	
**Menopausal status**				
premenopause	133 (50.0)	114 (48.7)	19 (59.4)	0.258
postmenopause	133 (50.0)	120 (51.3)	13 (40.6)	
**Imaging**				
**Rim enhancement**				
No	178 (66.7)	162 (68.9)	16 (50.0)	0.033
Yes	89 (33.3)	73 (31.1)	16 (50.0)	
**Peritumoral edema**				
No	163 (61.1)	145 (61.7)	18 (56.3)	0.553
Yes	104 (39.0)	90 (38.3)	14 (43.8)	
**BPE grade**				
minimal	143 (53.8)	125 (53.4)	18 (56.3)	0.144
mild	53 (19.9)	51 (21.8)	2 (6.3)	
moderate	43 (16.2)	35 (15.0)	8 (25.0)	
marked	27 (10.2)	23 (9.8)	4 (12.5)	
**Ipsilateral vascularity**				
Mean ± SD	2.0 ± 1.8	1.9 ± 1.7	3.0 ± 2.4	0.010
Median (IQR)	2.0 (1.0–3.0)	2.0 (0.0–3.0)	3.0 (1.0–5.0)	
**Immunohistochemical staining**				
**Subtype**				
luminal A	63 (23.8)	58 (24.9)	5 (15.6)	0.314
luminal B	124 (46.8)	106 (45.5)	18 (56.3)	
HER2 +	34 (12.8)	32 (13.7)	2 (6.3)	
Triple negative cancer (TNC)	44 (16.6)	37 (15.9)	7 (21.9)	
**Staging**				
0	13 (4.9)	13 (5.6)	0 (0.0)	0.001
I	77 (29.1)	70 (29.9)	7 (22.6)	
II	126 (47.6)	116 (49.6)	10 (32.3)	
III	45 (17.0)	33 (14.1)	12 (38.7)	
IV	4 (1.5)	2 (0.9)	2 (6.5)	
**Texture analysis**				
**Kurtosis**				
Mean ± SD	2.9 ± 0.7	2.9 ± 0.7	2.9 ± 0.8	0.802
Median(IQR)	2.8 (2.5–3.2)	2.8 (2.5–3.2)	2.7 (2.5–3.2)	
**Skewness**				
Mean ± SD	−0.1 ± 0.4	−0.1 ± 0.4	0.0 ± 0.4	0.031
Median (IQR)	−0.1 (−0.3–0.1)	−0.2 (−0.4–0.1)	−0.1 (−0.2–0.3)	
**Entropy**				
Mean ± SD	2.0 ± 0.6	2.0 ± 0.6	2.1 ± 0.5	0.359
Median (IQR)	1.9 (1.6–2.4)	1.9 (1.6–2.4)	2.0 (1.8–2.5)	
**Perfusion parameters**				
***Ktrans***				
**25percentile**				
Mean ± SD	0.2 ± 0.1	0.2 ± 0.1	0.2 ± 0.1	0.325
Median (IQR)	0.1 (0.1–0.2)	0.1 (0.1–0.2)	0.2 (0.1–0.2)	
**50percentile**				
Mean ± SD	0.3 ± 0.2	0.3 ± 0.2	0.3 ± 0.1	0.929
Median (IQR)	0.2 (0.2–0.3)	0.2 (0.2–0.3)	0.2 (0.2–0.4)	
**75percentile**				
Mean ± SD	0.4 ± 0.3	0.4 ± 0.3	0.4 ± 0.1	0.858
Median (IQR)	0.3 (0.2–0.5)	0.3 (0.2–0.5)	0.3 (0.2–0.5)	
**Mean**				
Mean ± SD	0.9 ± 9.7	1.0 ± 10.3	0.3 ± 0.1	0.962
Median (IQR)	0.2 (0.2–0.3)	0.2 (0.2–0.3)	0.2 (0.2–0.4)	
*Kep*				
**25percentile**				
Mean ± SD	0.3 ± 0.4	0.3 ± 0.4	0.3 ± 0.2	0.211
Median (IQR)	0.3 (0.2–0.4)	0.3 (0.2–0.4)	0.3 (0.2–0.4)	
**50percentile**				
Mean ± SD	0.6 ± 0.8	0.6 ± 0.9	0.5 ± 0.2	0.783
Median (IQR)	0.5 (0.4–0.6)	0.5 (0.4–0.6)	0.5 (0.4–0.6)	
**75percentile**				
Mean ± SD	0.9 ± 1.7	0.9 ± 1.8	0.8 ± 0.3	0.921
Median (IQR)	0.7 (0.5–0.9)	0.7 (0.5–0.9)	0.7 (0.6–0.9)	
**mean**				
Mean ± SD	0.7 ± 1.7	0.7 ± 1.8	0.6 ± 0.2	0.742
Median (IQR)	0.5 (0.4–0.7)	0.5 (0.4–0.7)	0.5 (0.5–0.6)	
*Ve*				
**25percentile**				
Mean ± SD	102.1 ± 1636.1	115.4 ± 1740.0	0.4 ± 0.1	0.197
Median (IQR)	0.4 (0.3–0.5)	0.4 (0.3–0.5)	0.4 (0.3–0.5)	
**50percentile**				
Mean ± SD	133.6 ± 2141.5	151.0 ± 2277.5	0.5 ± 0.2	0.681
Median (IQR)	0.5 (0.4–0.6)	0.5 (0.4–0.6)	0.5 (0.4–0.6)	
**75percentile**				
Mean ± SD	133.7 ± 2141.5	151.1 ± 2277.5	0.6 ± 0.2	0.960
Median (IQR)	0.6 (0.5–0.8)	0.6 (0.5–0.8)	0.6 (0.5–0.7)	
**mean**				
Mean ± SD	110.1 ± 1763.4	124.4 ± 1875.3	0.5 ± 0.2	0.611
Median (IQR)	0.5 (0.4–0.6)	0.5 (0.4–0.6)	0.5 (0.4–0.6)	

Of 32 patients who had disease recurrence, three died within 17 months after recurrence was diagnosed, and three showed disease progressions. All of these patients had distant metastases. Twenty five patients remained disease-free after appropriate adjuvant treatment – hormonal therapy, chemotherapy, or radiation therapy – or repeat surgery. Only one patient was unable to follow-up after disease recurrence diagnosis.

### Recurrence-associated factors

We assessed factors associated with disease recurrence according to the univariable and multivariable Cox proportional hazard regressions. Imaging parameters and clinical factors with p value less than 0.05 in univariable analysis were included as input parameters for multivariable analysis; pathologic stage, presence of rim enhancement, ipsilateral increased vascularity, skewness of texture analysis, and 25th percentile of Kep of perfusion parameters (Table 2). Tumor subtype was included in the final multivariable model despite not being statistically significant in univariable model because of its significant clinical relevance.

**Table 2:** Univariable Cox proportional hazards regression.

	HR (95% CI)	p value
**Clinicopathologic Factors**		
Patient age (≤40)	0.66 (0.27–1.59)	0.352
Menopausal status	0.71 (0.35–1.44)	0.341
Subtype		0.328
Luminal A	Reference	
Luminal B	1.67 (0.62–4.49)	0.309
Her2 +	0.83 (0.18–3.86)	0.808
TNC	2.44 (0.78–7.62)	0.124
Staging		0.002
0	0.37 (0.02–7.02)	0.504
I	1.20 (0.45–3.19)	0.713
II	Reference	
III	3.64 (1.54–8.58)	0.003
IV	9.31 (2.20–39.43)	0.003
**Morphologic features**		
**Rim enhancement**		
No	Reference	
Yes	2.02 (1.01–4.05)	0.047
**Peritumoral edema**		
No	Reference	
Yes	1.32 (0.66–2.65)	0.438
BPE grade		0.155
Minimal	Reference	
Mild	0.37 (0.09–1.45)	0.154
Moderate	1.75 (0.75–4.09)	0.194
Marked	1.63 (0.56–4.76)	0.376
**Ipsilateral vascularity**		
<3	Reference	
≥3	2.85 (1.41–5.74)	0.003
**Quantitative parameters**^**†**^		
***Texture analysis***		
**Kurtosis**		
<3.61	Reference	
≥3.61	1.79 (0.70–4.55)	0.223
**Skewness**		
<0.29	Reference	
≥0.29	2.97 (1.37–6.43)	0.006
**Entropy**		
<1.82	Reference	
≥1.82	2.00 (0.90–4.44)	0.089
***Perfusion parameters***		
Ktrans		
**25percentile**		
<0.16	Reference	
≥0.16	1.53 (0.75–3.13)	0.244
**50percentile**		
<0.42	Reference	
≥0.42	0.28 (0.05–1.47)	0.132
**75percentile**		
<0.58	Reference	
≥0.58	0.11 (0.01–1.87)	0.126
**mean**		
<0.43	Reference	
≥0.43	0.12 (0.01–2.02)	0.14
**Kep**		
**25percentile**		
<0.20	Reference	
≥0.20	3.12 (1.01–9.68)	0.049
**50percentile**		
<0.61	Reference	
≥0.61	0.65 (0.27–1.57)	0.336
**75percentile**		
<0.55	Reference	
≥0.55	2.26 (0.82–6.25)	0.116
**mean**		
<0.45	Reference	
≥0.45	1.93 (0.84–4.46)	0.123
**Ve**		
**25 percentile**		
<0.20	Reference	
≥0.20	6.99 (0.41–119.97)	0.18
**50 percentile**		
<0.55	Reference	
≥0.55	1.65 (0.81–3.39)	0.169
**75 percentile**		
<0.73	Reference	
≥0.73	0.56 (0.23–1.35)	0.195
**mean**		
<0.53	Reference	
≥0.53	1.65 (0.80–3.38)	0.172

^**†**^Optimal cut-off value of each quantitative parameter was determined using maximally selected rank statistics.

Model A was composed of clinicopathologic factors, including tumor subtype and pathologic stage. In model A, stage III and IV showed significant correlation with disease recurrence. The higher the pathologic stage was, the greater the correlation with disease recurrence was. Model B was composed of morphologic features including rim enhancement and ipsilateral vascularity, and clinico-pathologic factors including pathologic stage and tumor subtype. There was a significant correlation with the recurrence of disease in cases with increased ipsilateral vascularity (≥3), whereas rim enhancement showed no association with disease recurrence in model B. Model C was composed of quantitative parameters including skewness of texture analysis, 25th percentile of Kep of perfusion parameters and clinico-pathologic factors including pathologic stage and tumor subtype. In model C, higher skewness of texture analysis and higher Kep 25th percentile value showed significant correlation with disease recurrence. Finally, Model D included all clinicopathologic factors, morphologic features and quantitative MR parameters; pathologic stage, tumor subtype, ipsilateral vascularity, rim enhancement and skewness of texture analysis and 25th percentile of Kep of perfusion parameters. In model D, increased ipsilateral vascularity (≥3) and higher positive skewness of texture analysis were independently associated with disease recurrence (Table 3).

**Table 3:** Multivariable Cox proportional hazards regression.

	adjusted HR (95% CI)	p value
**Model A**		
**Subtype**		
Luminal A	Reference	
Luminal B	1.46 (0.54–3.95)	0.462
Her2 +	0.87 (0.19–4.01)	0.856
TNC	1.57 (0.47–5.22)	0.46
**pTNM**		
0	0.51 (0.03–9.79)	0.652
I	1.23 (0.46–3.28)	0.673
II	Reference	
III	3.63 (1.53–8.58)	0.003
IV	7.89 (1.79–34.68)	0.006
**Model B**		
**Subtype**		
Luminal A	Reference	
Luminal B	1.40 (0.51–3.83)	0.518
Her2 +	0.57 (0.12–2.71)	0.476
TNC	1.43 (0.42–4.85)	0.566
**pTNM**		
0	1.96 (0.10–38.63)	0.658
I	1.56 (0.08–30.19)	0.768
II	Reference	
III	5.46 (0.29–104.18)	0.259
IV	9.28 (0.37–231.50)	0.175
**Rim enhancement**		
No	Reference	
Yes	1.58 (0.74–3.38)	0.234
**Ipsilateral vascularity**		
<3	Reference	
≥3	2.67 (1.28–5.60)	0.009
**Model C**		
**Subtype**		
Luminal A	Reference	
Luminal B	1.84(0.66–5.15)	0.246
Her2 +	0.81(0.17–3.80)	0.787
TNC	2.56(0.73–8.98)	0.141
**pTNM**		
0	0.58(0.03–11.53)	0.723
I	1.45(0.53–3.96)	0.470
II	Reference	
III	3.59(1.44–9.00)	0.006
IV	3.86(0.79–18.85)	0.095
**Texture analysis: Skewness**		
<0.29	Reference	
≥0.29	2.85(1.17–6.94)	0.021
**Kep: 25perc**		
<0.20	Reference	
≥0.20	3.84(1.16–12.72)	0.028
**Model D**		
**Subtype**		
Luminal A	Reference	
Luminal B	1.71 (0.60–4.88)	0.317
Her2 +	0.53 (0.11–2.61)	0.437
TNC	1.96 (0.54–7.06)	0.306
**pTNM**		
0	0.85 (0.04–16.96)	0.913
I	1.46 (0.53–4.05)	0.465
II	Reference	
III	3.45 (1.37–8.67)	0.008
IV	3.27 (0.67–16.00)	0.144
**Rim enhancement**		
No	Reference	
Yes	2.09 (0.92–4.76)	0.08
**Ipsilateral vascularity**		
<3	Reference	
≥3	2.71 (1.25–5.86)	0.011
**Texture analysis: Skewness**		
<0.29	Reference	
≥0.29	3.21 (1.26–8.23)	0.015
**Kep: 25perc**		
<0.20	Reference	
≥0.20	2.66 (0.82–8.67)	0.105

Comparison of C index of each model revealed that model D, including all clinicopathologic and imaging factors, showed the highest C index (0.825 [95% CI: 0.755–0.896]) with excellent discrimination for high risk group of recurrence. Model B also showed excellent discriminative power for high risk group of recurrence with C index of 0.800 (95% CI: 0.724–0.876). Model C also showed acceptable discrimination with C index of 0.752 (95% CI: 0.655–0.848). When the C index of model B, C, D were compared to that of model A, model D showed the highest C index and barely escaped being statistically significant at the 5% risk level (p = 0.052). Model B and C also showed higher C index than that of model A, although without significance (Table 4).

**Table 4:** C statistics of multivariable Cox proportional hazards regression models.

Model	Harrell’s C index (95% CI)	p value
Model A	0.698 (0.590–0.805)	Reference
Model B	0.800(0.724–0.876)	0.128
Model C	0.752(0.655–0.848)	0.464
Model D	0.825 (0.755–0.896)	0.052

### Survival analysis

**Figure 1:**
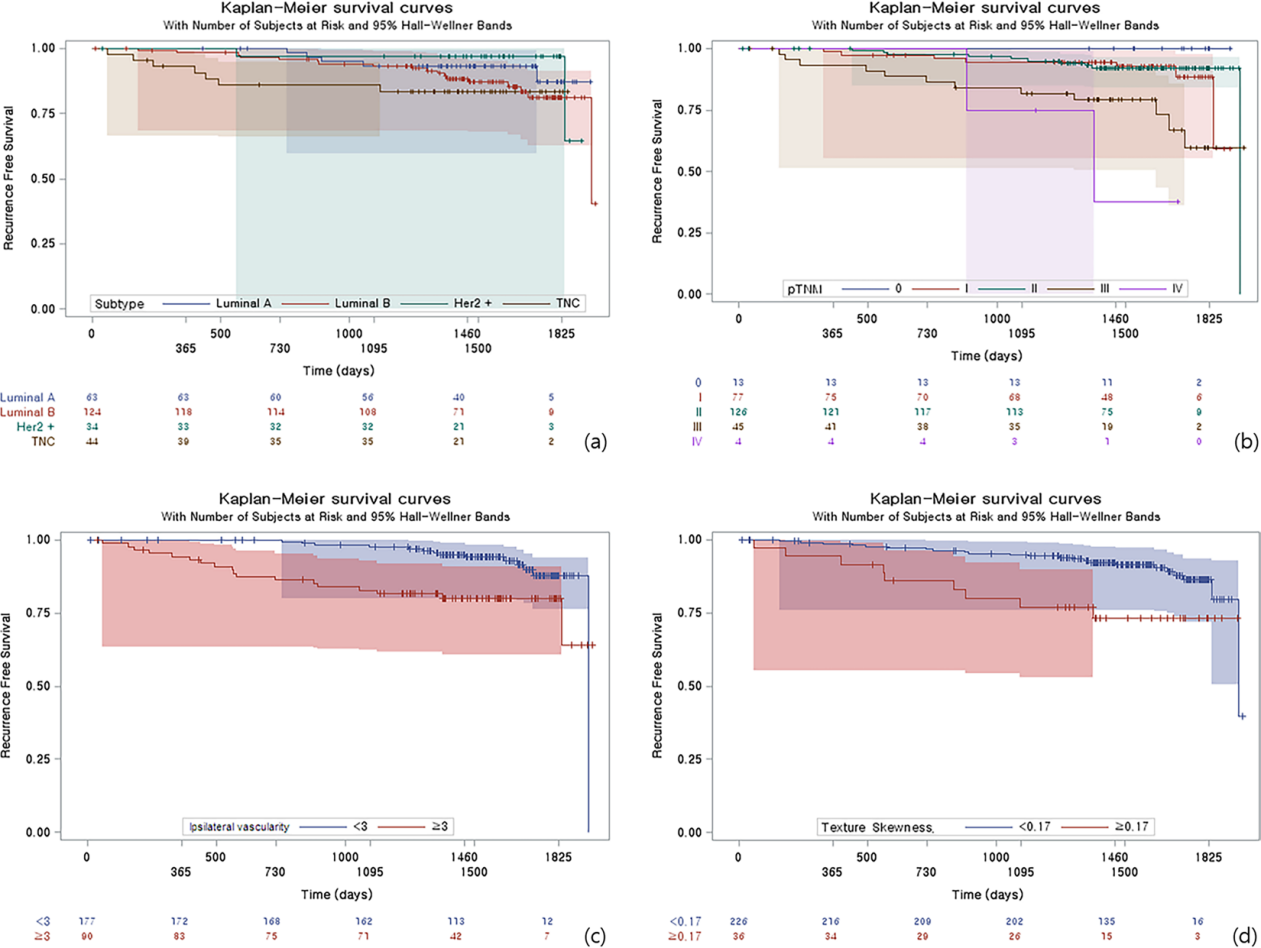
Kaplan-Meier survival curves according to (**a**) tumor subtype, (**b**) pathologic stage, (**c**) ipsilateral vascularity, (**d**) skewness. Higher pathologic stage (over stage II, p < 0.001), increased ipsilateral vascularity (≥3, *p* < 0.002), higher positive skewness (≥0.29 *p* = 0.005) were associated with worse DFS.

Of the imaging parameters associated with disease recurrence, increased ipsilateral vascularity and skewness of texture analysis were associated with worse DFS in Kaplan-Meier analysis. Patients with increased vascularity (≥3) on MRI had shorter recurrence intervals (*p* = 0.002) than those without increased vascularity (<3) on MRI. Patients with higher positive skewness (*p* = 0.005) in texture analysis showed lower DFS. We observed lower DFS in patients with triple negative subtype cancer compared to those with other subtypes, but the difference was not significant (*p* = 0.243) (Fig. 1).

In addition to MRI parameters, the pathologic stage was associated with worse DFS (*p* = 0.002). In other words, higher pathologic stage of initial breast cancer was associated with worse DFS.

## Discussion

With the advancement of MR technology, it has become easier to obtain various qualitative parameters. Perfusion parameters and texture analysis are representative quantitative parameters that are being used to predict disease prognosis. In this study, we tried to predict disease recurrence using both qualitative morphologic features of MRI and quantitative MR parameters. Of several multivariable models, the comprehensive model showed that, increased ipsilateral vascularity and skewness of texture analysis are associated with disease recurrence independently with the best discriminative performance.

Increased vascularity of the tumor-involved breast is related to neoangiogenesis in tumors. Increase in the number of vessels around the tumor contribute to hematogenous spread of tumor cells and distant metastasis of tumors^20^. For this reason, increased vascularity around a tumor may be associated with disease recurrence and worse DFS, as shown in the present study. Also, increased vascularity always showed significant association with disease recurrence in every multivariable model. Increased vascularity due to the tumor can affect other MRI parameters related to tumor vascularity, including perfusion or texture parameters^13,19^. However, texture and perfusion parameters were associated with disease recurrence independent of increased ipsilateral vascularity in present study.

Generally higher entropy, kurtosis, and positive skewness of the tumor in DCE-MRI suggest poor prognosis in texture analysis^21,22^. Especially for skewness, it represents a measure of asymmetry of probability distribution. Higher positive skewness could mean long right-sided tail with lower mean value in histogram. In our study, it could mean that lower mean signal intensity (SI) in the fat-saturated contrast enhanced T1 weighted images (WI). Low SI of tumor in fat-saturated contrast enhanced T1WI may suggested the situation such tumor necrosis. Considering tumor necrosis is often accompanied in high grade tumor with poor prognosis, higher positive skewness may suggest poor prognosis.

As shown in result, multivariable Cox proportional hazard model using clinicopathologic factors and both morphologic feature and quantitative parameters showed the highest and excellent discriminative ability (C index 0.825, [95% CI: 0.755–0.896]) among multivariable models. The C index of the comprehensive model was higher than that of the model with clinico-pathologic factors alone with statistical relevance. Also, although not statistically significant, the comprehensive model showed higher C index than the model with morphologic feature and clinico-pathologic factors. In other words, applying advanced MR parameters to the previously known clinico-pathological predictors and morphologic imaging characteristics could be more helpful to predict disease recurrence.

The imaging parameters which were associated with disease recurrence, also showed association with lower DFS in Kaplan-Meier analysis. Some previous studies suggested that patients with shorter interval between surgery and first relapse had shorter overall survival in breast cancer^23–25^. In other words, DFS itself is an important prognostic factor that can predict overall survival in recurred patients. In our study, recurrent diseases were confirmed mostly within 3 years (mean 31 months, median 28.5 months) after first preoperative MRI. Of 32 patients with disease recurrence, three died within 17 months. Although it is difficult to analyze overall survival due to the small number of expired cases in our study, it would be possible to obtain imaging parameters related to overall survival with more patients and a longer follow-up period.

There are a number of limitations of the present study. It was a retrospective, single-center study with a small number of patients with disease recurrence. The disease recurrence event number was much smaller than the number of total patients. This can weaken the representativeness of results. In addition, due to differences in follow-up period after surgery, disease recurrence may not have been fully investigated at the time of review. Also, basic information about the tumor, such as other morphologic features following the BI-RADS lexicon and kinetic curve pattern, was not included in the present study, despite being possible prognostic factors of disease recurrence.

We investigated morphologic features, quantitative MR parameters and clinicopathologic factors to predict disease recurrence in breast cancer patients. The present study analyzed multiple imaging prognostic factors more comprehensively that has been previously performed. We found that higher pathologic stage, increased ipsilateral vascularity and higher positive skewness of texture analysis were associated with disease recurrence independent of tumor subtype, and using quantitative MR parameters in addition to clinicopathologic factors and morphologic features could be helpful to predict disease recurrence more accurately. We expect that these results will help physicians predict the prognosis of breast cancer patients and choose appropriate treatment methods more easily, and ultimately will contribute to improving survival of breast cancer patients.

## Materials and Methods

### Patients

This study was approved by the Institutional Review Board of Seoul St. Mary’s Hospital. The requirement for informed consent was waived due to its retrospective nature and it was confirmed by IRB of our institution. Investigations were carried out as per the rules of the Declaration of Helsinki of 1975, revised in 2013.

**Figure 2:**
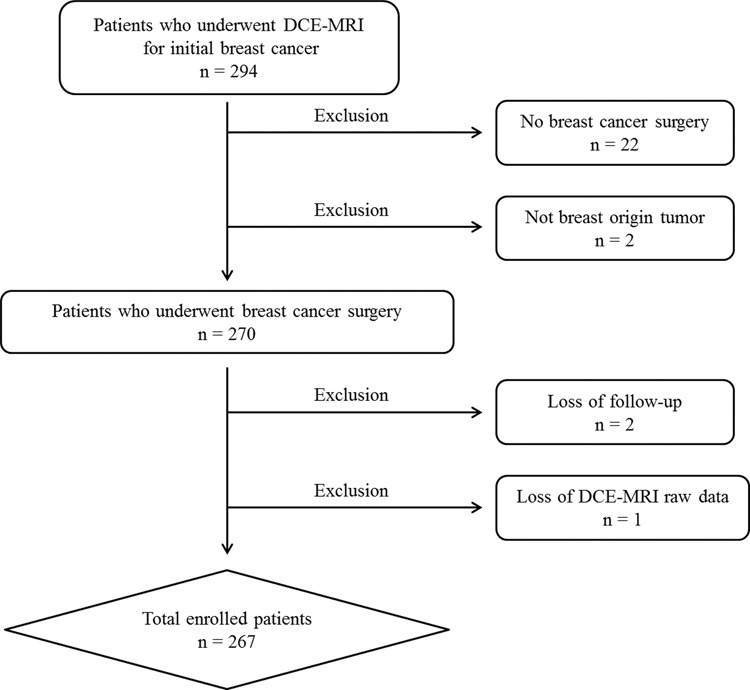
Study population with exclusion criteria.

A total of 294 breast cancer patients who underwent breast DCE-MRI between February 2014 and May 2016 was included in the study sample. Of 294 patients, 22 who had not undergone surgery for breast cancer were excluded. Two patients with cancers that were not breast cancer were also excluded. Of the remaining 270 patients, two who were lost to follow-up for more than one year and one without raw MR data were excluded. Finally, 267 patients were enrolled in our study (Fig. 2). All enrolled patients were treated according to standard treatment guidelines for breast cancer. Only one lesion was analyzed per patient. In cases of bilateral or multiple breast cancers, the largest lesion was analyzed as a target lesion.

### Clinicopathologic information

We reviewed the medical records of all 267 patients. Clinical information comprised patient age, menopausal status, date of pretreatment MRI scanning, and date of last visit to the outpatient clinic. For calculation of disease-free survival, we considered the date of pretreatment MRI scanning to be the first day of diagnosis. Generally, MRI scanning was performed 10 to 14 days after biopsy. Pathologic information for initial breast cancer was obtained from surgical specimens, including hormonal receptor status, Ki-67 index, and presence of lymphovascular invasion. Pathologic stage was determined based on the AJCC 7th edition.

Cancer recurrence was defined as newly diagnosed ipsilateral or contralateral breast cancer or distant metastasis after surgery. The day of confirmed recurrent cancer on biopsy was considered the date of recurrence. When biopsy data were not available, the date of recurring lesion detection by imaging scanning such as positron emission tomography (PET), bone scan, or computed tomography (CT) was considered the relapse date.

### MRI protocol

MRI examinations were performed with patients in the prone position using a Magnetom Verio 3 T system (Siemens Healthcare, Erlangen, Germany) and a dedicated eight-channel phase-array coil. Images were obtained using the following sequences: (1) axial turbo spin-echo T2-weighted imaging (T2WI) with TR/TE of 4530/93 msec, flip angle of 80°, field of view (FOV) 320 × 320 mm^2^, matrix size of 576 × 403, slice thickness of 4 mm, and acquisition time of 2 min 28 sec; (2) pre-contrast T1-weighted three-dimensional (3D) volumetric interpolated breath-hold examinations (3D VIBE) with TR/TE of 2.7/0.8 msec, FOV of 320 × 320 mm^2^, matrix size of 256 × 192, slice thickness of 2 mm with various flip angles (2°, 6°, 9°, 12°, 15°), and acquisition time of 2 min 15 sec to determine tissue T1 relaxation time prior to the arrival of contrast agent; (3) dynamic contrast-enhanced axial T1-weighted imaging (T1WI) with fat suppression with TR/TE of 2.5/0.8 msec, flip angle of 10°, slice thickness of 2.0 mm, and acquisition time of 5 min 30 sec (temporal resolution 6 sec) following an intravenous bolus injection of 0.1 mmol/kg gadobutol (Gadovist, Schering, Berlin, Germany) followed by a 20 ml saline flush; and (4) delayed axialT1-weighted 3D VIBE with TR/TE of 4.4/1.7 msec, flip angle of 10°, slice thickness of 1.2 mm, FOV of 340 mm, and matrix size of 448 × 358 to evaluate the overall extent of tumor.

### Morphologic analysis

Two breast radiologists with 5 years and 20 years of experience in breast imaging retrospectively reviewed pretreatment breast MRI using a picture archiving and communication system (PACS) (Maroview 5.4; In nitt, Seoul, Korea) and workstation monitor.

#### Enhancement pattern and background parenchymal enhancement (BPE)

The internal enhancement pattern (rim or non-rim) of the tumor and BPE degree (minimal/mild or moderate/marked) were analyzed following the guidelines of the fifth edition of the Breast Imaging Reporting and Data System (BI-RADS) MRI lexicon^26^. Cases of cancer expressed as non-mass enhancement only were considered negative for rim enhancement.

#### Ipsilateral vascularity

**Figure 3:**
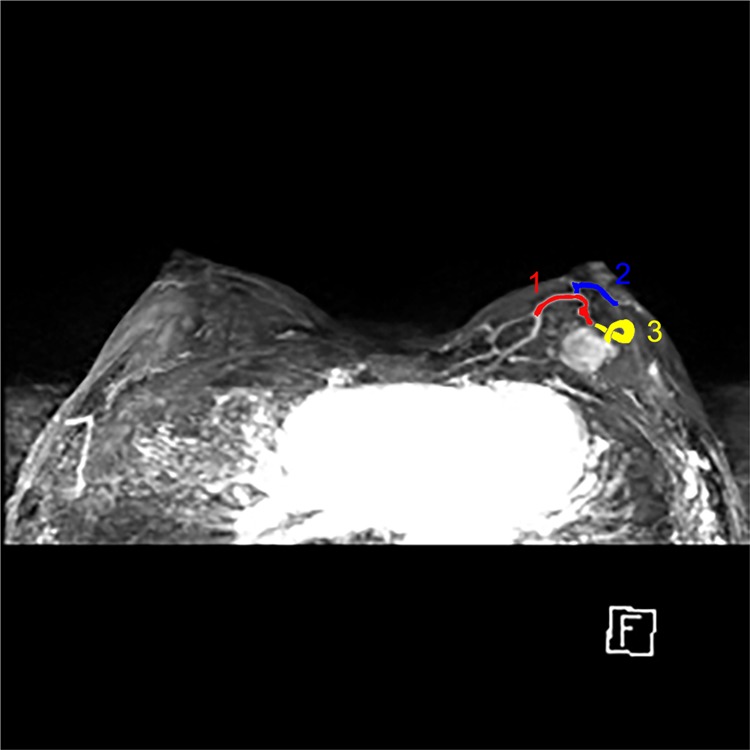
MIP images show prominent vessels of left breast with enhancing tumor at mid upper portion of left breast. According to our criteria, the number of vessels counted is three.

The number of vessels around the tumor was measured in maximum-intensity-projection (MIP) reconstruction views to reduce confusion caused by BPE. Only vessels 3 cm or longer in length and 2 mm or larger in maximal transverse diameter were recognized as target vessels. We counted the number of vessels visualized in the breast containing the index cancer (Fig. 3). In cases of bilateral or multiple breast cancers, the number of vessels in the breast that contained the largest tumor were counted^13^.

#### Peritumoral edema

**Figure 4:**
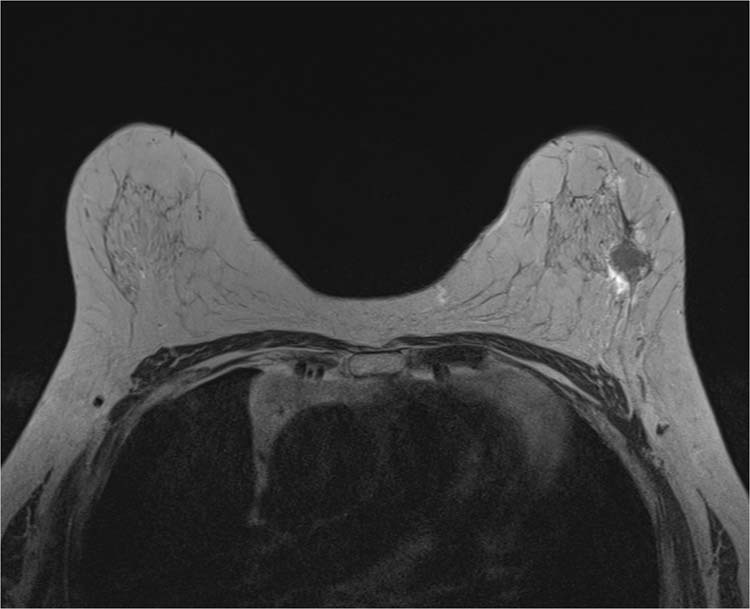
On axial T2-weighted image, high signal intensity which is defined as peritumoral edema, is noted at posterior aspect of breast tumor in left upper outer breast.

Peritumoral edema was defined as increased signal intensity around the tumor or high signal intensity that is similar to water or vessel signal intensity, posterior to the tumor in the prepectoral area in T2-weighted images. Peritumoral edema was evaluated by visual evaluation on the PACS system by both readers (Fig. 4)^15,16^.

### MR parameters

#### Perfusion parameters

**Figure 5:**
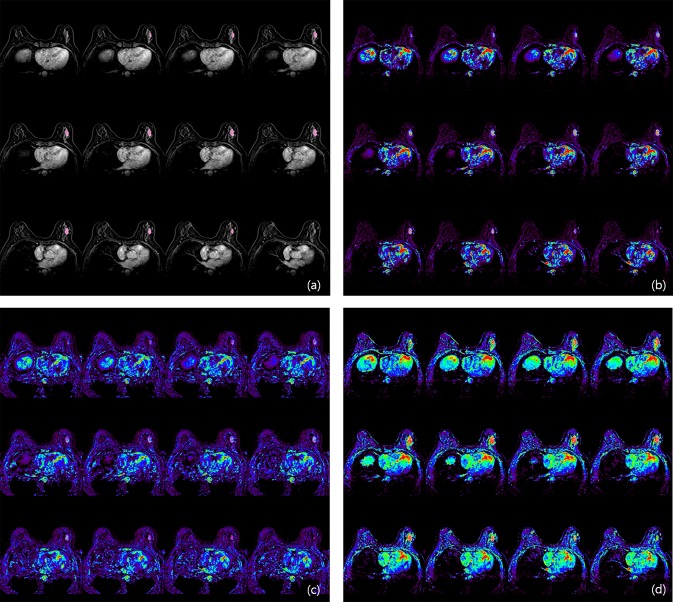
Evaluation of perfusion parameter. (**a**) On Fat-saturated T1-weighted images, tumor segmentation was performed with semi-automatic tool (magic wand tool). After applying tumor segmentation on Ktrans map (**b**), Kep map (**c**) and Ve map (**d**), perfusion parameters from each map were calculated.

A standard Tofts model was used to evaluate perfusion parameters on DCE-MRI. Methods used to analyze perfusion parameters were described in a previous study (Fig. 5)^27^.

#### Texture analysis parameters

**Figure 6:**
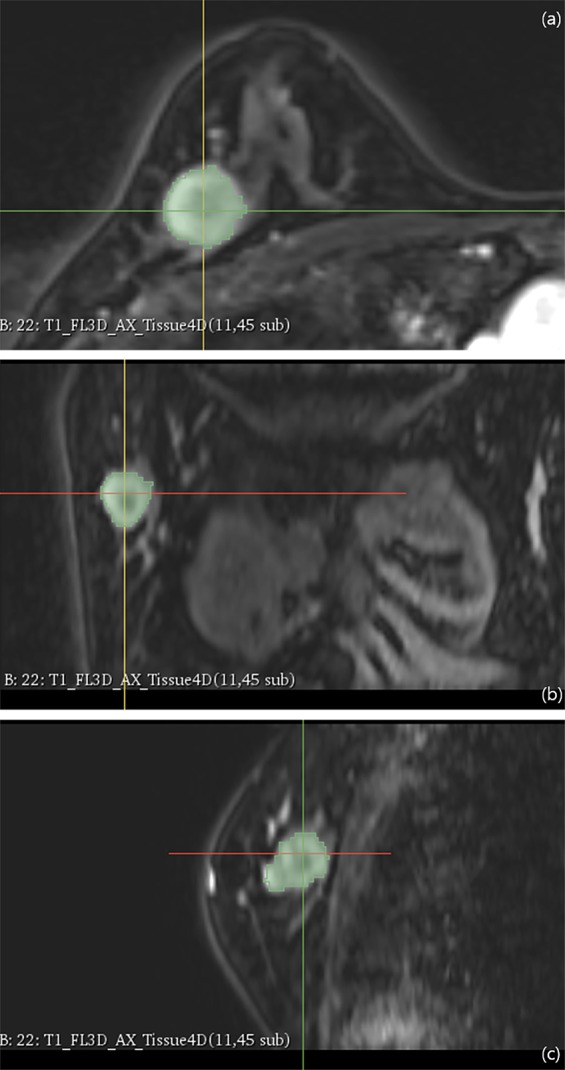
Texture analysis using 3D slicer. After tumor segmentation in the axial (**a**), coronal (**b**) and sagittal planes (**c**), texture parameters of tumor were calculated.

For texture analysis, we used free and open source software package for visualization and medical image computing (3D slicer, ver. 4.8.0; available at: https://slicer.org/), with dynamic contrast-enhanced T1-weighted images with fat saturation derived from the PACS system. Contrast-enhanced T1-weighted images acquired 80 seconds after contrast material injection were assessed. After selection of the enhancing tumor, semiautomatic tumor segmentation was performed, and 19 texture features were extracted automatically. Among these 19 features, we selected entropy, skewness, and kurtosis to evaluate tumor heterogeneity. In cases of multifocal, multicentric, or bilateral cancer, texture analysis was performed for the largest tumor (Fig. 6).

### Statistical analysis

Using univariable and multivariable Cox proportional hazard regression analyses, we estimated hazard ratios with 95% confidence intervals (CI)s for disease recurrence according to imaging parameters and clinicopathological factors. First, univariable Cox proportional hazard regression analysis was performed for each parameter. Then, multivariable Cox proportional hazard regression analysis was performed using parameters with *p* < 0.05 in univariable analysis and other parameters of known clinical relevance. Stepwise selection was applied to control multicollinearity and determine the final model^28,29^.

Because tumor subtype may affect imaging features of tumors on MRI, multivariable models were built to exclude the effects of clinical factors on imaging parameters. Multivariable models were composed in four ways: clinico-pathologic factors alone, qualitative parameters with clinico-pathologic factors, quantitative parameters with clinico-pathologic factors, and qualitative and quantitative imaging parameters, and clinico-pathologic factors. In multivariable analysis, parameters with *p* < 0.05 were considered statistically significant.

The C statistics was used for discrimination of high and low risk patients for disease recurrence. The C statistics value ranges from 0.5 (no discrimination) to 1.0 (perfect discrimination). According to Hosmer and Lemeshow, all values were interpreted as acceptable discrimination (0.7–0.8), excellent discrimination (0.8–0.9), and outstanding discrimination (≥0.9). The C statistic values of each model of multivariable regression analysis were compared^30^.

Missing data were deleted pairwise, minimizing loss of data by using all available cases for each analysis (missing rate: less than 6.5% of kinetic parameters and less than 3% of otherwise). To determine optimal cutoff values of imaging parameters to predict recurrence, we used maximally selected rank statistics.

Kaplan-Meier estimates were used for comparison of disease-free survival (DFS) between the recurrent group and non-recurrent group using imaging parameters and clinicopathological factors that were significant in univariable Cox proportional hazards regression. Survival differences were compared using log rank test.

Statistical analyses were performed using SAS version 9.4 (SAS Institute, Cary, North Carolina, USA) and the R “maxstat” package in R version 2.15.3 (R Foundation, Vienna, Austria; http://www.R-project.org).
